# Separating Overlapping Birdsongs Enhances the Reliability of Avian Vocal Activity Analysis

**DOI:** 10.1002/ece3.73648

**Published:** 2026-05-11

**Authors:** Jie Wang, YanChao Lai, Xuehan Wang, Jinhui Li, Yanqin Wang, Shegang Shao, Minmin Yuan

**Affiliations:** ^1^ School of Electronics and Communication Engineering Guangzhou University Guangzhou China; ^2^ Guangdong Provincial Highway Construction Limited Company Guangzhou China; ^3^ Research Institute of Highway Ministry of Transport Beijing China

**Keywords:** bird behavioral responses, bird song separation, climate impact, passive acoustic monitoring (PAM), vocal activity, vocal activity rate

## Abstract

Bird vocalizations are important indicators in ecological research, used to assess population size and habitat use. Long‐term monitoring of changes in bird vocalizations can also reveal patterns of environmental change affecting birds. However, recordings obtained through passive acoustic monitoring often contain overlapping calls from multiple bird species and background noise interference, reducing the reliability of automated identification and ecological analysis. This study uses an improved deep learning algorithm that dynamically processes mixed bird calls of uncertain numbers in the field through a counter and decoder, and employs three different strategies to verify the impact of the separation front‐end on classification performance. We conducted a field study in the Huangmaohai Cross‐Sea Channel area in southern China to verify the effectiveness of the separation algorithm. The study shows that separation effectively filters out misclassifications caused by noise or calls from other species, correcting biases in the original monitoring. By studying the diurnal variation patterns of calls of the common diurnal bird, the White Wagtail, and the nocturnal bird, the Black‐crowned Night Heron, we demonstrate that separation can effectively eliminate false activity peaks of the White Wagtail in the evening and restore the masked nocturnal activity rhythms of the Black‐crowned Night Heron. Separation also helps to reveal the suppression of bird vocalizations by high temperatures and precipitation, improving the statistical sensitivity of environmental factors. Furthermore, the separation results revealed the seasonal vocalization patterns of birdsong in the region. This study provides important support for sound analysis in multi‐species resonance scenarios during ecological monitoring.

## Graphical abstract

Monitoring avian vocalizations plays a crucial role in biodiversity assessment and ecological management, yet overlapping acoustic signals in natural environments often hinder accurate recognition and estimation. This study addresses that challenge by developing and validating a deep learning‐based source separation framework specifically designed for multi‐species bird communities.

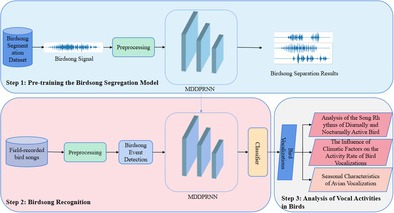

## 1 Introduction

In ecological research and biodiversity monitoring, birds are widely regarded as important ecological indicator species due to their high sensitivity to environmental changes (Peck et al. 2014). Avian vocal activity can reflect their social interactions, which is crucial for understanding their life history, social behavior, and ecological adaptations (Pérez‐Granados and Schuchmann 2023; Alonso et al. 2021). Temporal variations in vocal activity during the breeding season are typically associated with breeding status or territory establishment and defense (Voigt et al. 2021; Derrickson 1988), dynamically reflecting the reproductive state of birds (Flis 2023; Schaaf et al. 2023). For example, male Great Curassows exhibit maximum vocal intensity during territory establishment and early mate attraction processes. In the later stages of the breeding season, increased focus on foraging, chick care, and nest protection leads to a decrease in vocal activity (Baldo and Mennill 2011). Simultaneously, avian vocal activity is also influenced by climatic factors, which typically reflects adaptations to environmental changes (Wang et al. 2022; Ramírez et al. 2024). For instance, the dawn chorus frequency of Alström's Warblers and Brown‐flanked Bush Warblers decreases significantly as temperature rises (Puswal et al. 2021). Diurnal birds, such as *Crypturellus undulatus* and *Ortalis canicollis* , exhibit the highest vocal activity during the dry season, which declines with the arrival of the rainy season, offering insights into how avian vocal activity responds to climate change (Pérez‐Granados and Schuchmann 2021a). Weather also affects vocal behavior; rain may negatively impact vocal intensity by reducing communication between conspecifics through acoustic masking (Vokurková et al. 2018; Lengagne and Slater 2002).

Vocal activity rate (VAR) reflects the number of vocalizations produced per unit of time and is a key metric for the quantitative analysis of vocal temporal dynamics (Oppel et al. 2014). VAR not only reflects individual physiological states and behavioral patterns but is also closely related to population health, reproductive success, and habitat environment (Godfrey 2003; Hiebert et al. 1989). For example, a high VAR typically indicates species activity, abundant resources, and a stable ecological environment (Gil and Gahr 2002); whereas a decline in VAR may imply habitat degradation, food scarcity, or increased anthropogenic disturbance (Shannon et al. 2016). Furthermore, based on the density limitation hypothesis of songbirds, VAR can predict bird abundance around recording devices, and by assessing individual vocalizations over time, this metric can be used to monitor population density dynamics (Pérez‐Granados et al. 2019).

Studying avian vocal activity requires long‐term data, but traditional methods of obtaining continuous recordings are labor‐intensive and resource‐heavy, and manual recording may disturb target species (Marques et al. 2013). With advancements in recording and storage devices, passive acoustic monitoring (PAM) technology has been widely applied in various bird monitoring fields due to its ability to rapidly and automatically collect massive spatiotemporal data while minimizing potential biases from field observers (Hou et al. 2022; Ma and Fan 2023). Meanwhile, PAM combined with automated acoustic recognition technology has also been widely used to calculate avian vocal activity rate (Maithripala et al. 2024). However, PAM recordings often contain numerous mixed sound sources, such as overlapping calls from multiple bird individuals or species. The mixture of these non‐target sound sources greatly increases the difficulty of subsequent identification and analysis, making bird identification, call event counting, and vocal activity analysis susceptible to interference, which in turn affects judgments regarding ecological environmental changes (Gibb et al. 2019). Therefore, it is necessary to effectively separate bird song signals from complex ecological audio to improve the accuracy of subsequent identification and VAR analysis results. Because achieving low‐cost, large‐scale, long‐term environmental monitoring typically requires field recording equipment to be configured with a single microphone (Hill et al. 2018), bird song acoustic separation is usually studied focusing on single‐channel audio. In recent years, single‐channel sound source separation techniques based on deep learning have been the primary approach adopted. However, existing bird song separation research still faces significant limitations when dealing with complex wild soundscapes: the time‐domain DPTTNet proposed by Zhang et al. (2022), although significantly improving inference efficiency through a dual‐path network, presupposes a fixed 2‐source output channel architecture, capable of separating only two bird songs, making it difficult to adapt to scenarios with dynamic changes in the number of field sound sources; BACPPNet based on frequency‐domain masking (Xie et al. 2024) utilizes dilated convolutions to enhance long‐term capture capabilities and extends to the separation of 3 bird songs, but the model struggles to generalize to mixed audio with unknown quantities; while the unsupervised MixIT framework introduced by Denton et al. (2022) reduces dependence on annotated data, its “over‐separation” strategy leads to redundant output channels, requiring a preset number of output channels and failing to capture the number of birds in the mixed audio, necessitating complex post‐processing algorithms to filter effective sound sources.

**FIGURE 1:**
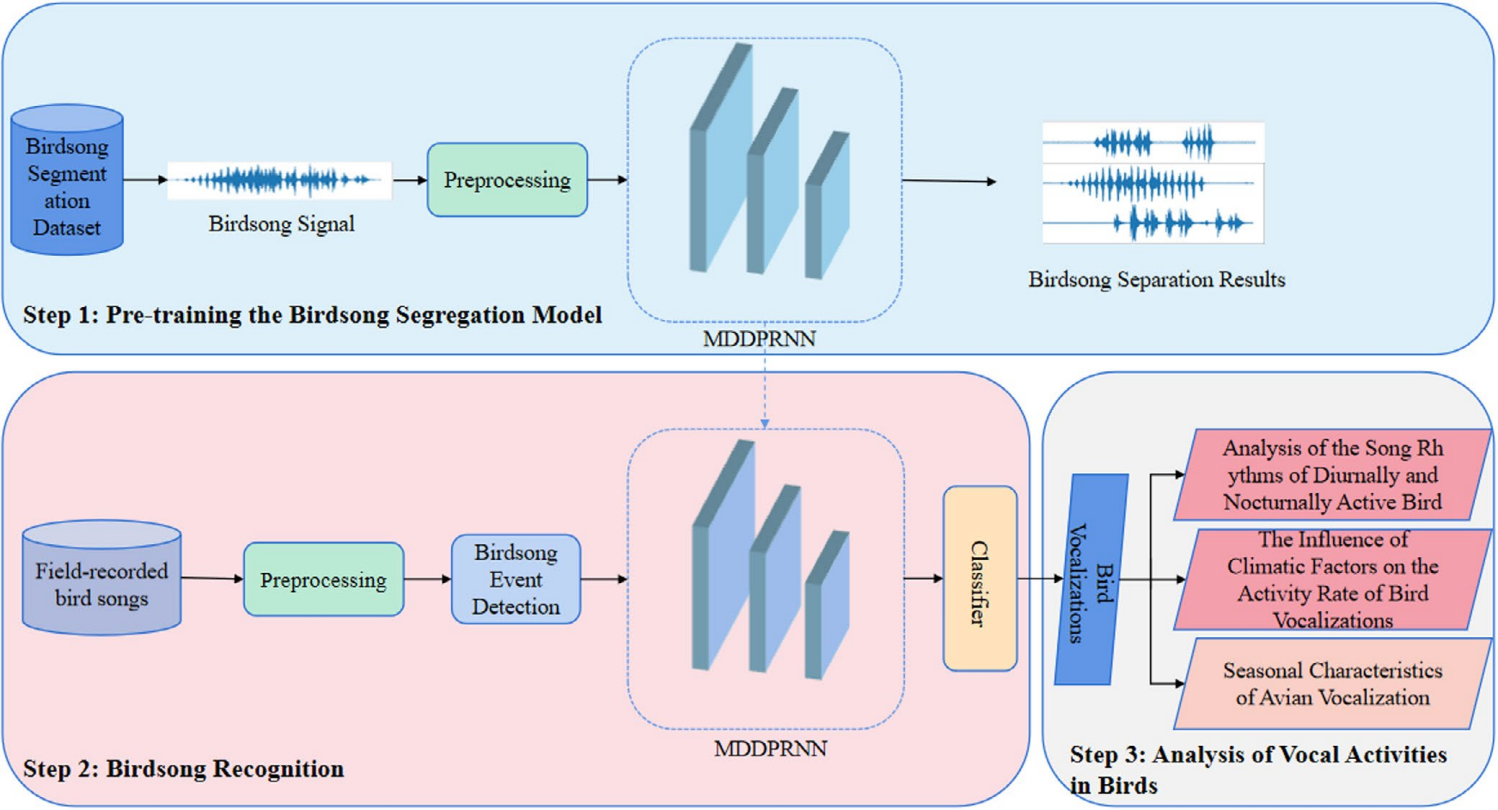
Overall technical flowchart.

In summary, current mainstream methods for bird sound separation require the prespecification of a fixed number of mixed sources and are incapable of separating mixtures where the number of sources is unknown. Furthermore, existing models are often designed for simulated rather than field‐collected mixed data, and no research has yet addressed the complex reality of multiple bird species, multiple sound sources, and unknown quantities in real‐world wild environments. Therefore, this paper proposes a bird sound separation framework designed for an unknown number of mixed bird species. Using the re‐identified bird sound data obtained after separation, this study conducts relevant research and analysis on the vocal activity of the nocturnal Black‐crowned Night Heron and the diurnal White Wagtail, which are common in the Huangmaohai Sea‐crossing Channel area. The aim is to verify whether separation improves the recognition rate of overlapping bird songs and enhances the reliability of ecological analysis regarding avian vocal activity. The contributions and overall technical route of this paper are illustrated in Figure 1 and summarized as follows: (1) A bird sound separation dataset was constructed, a counter module was improved based on the frequency characteristics of bird sounds, and a multi‐bird signal separation model that does not require determining the mixing quantity was established; (2) Field recordings of mixed bird audio from the Huangmaohai Sea‐crossing Channel area were preprocessed and input into the pre‐trained separation model, and then fed into a classifier for bird sound recognition; (3) Based on the recognition results, avian vocal activity was derived to analyze the vocal activity characteristics of typical nocturnal and diurnal birds in the Huangmaohai region, as well as to analyze the influence of climatic factors on the vocal activity rate and seasonal variations in vocalizations.

## 2 Materials and Methods for Bird Sound Separation

### 2.1 Data Collection and Recording Settings

**FIGURE 2:**
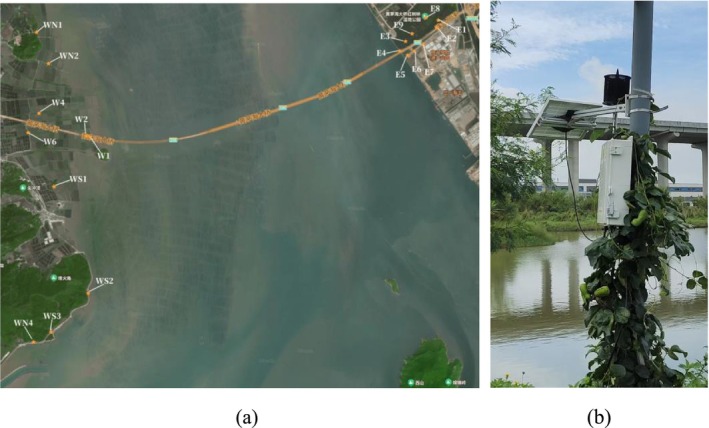
(a) Wildlife recorder installation. (b) Map illustrating locations where birdsong was recorded in Huangmaohai Sea‐crossing Channel.

The Huangmaohai Sea‐crossing Channel connects Jinwan District in Zhuhai City and Taishan City in Guangdong Province. A total of 16 sites were selected within the project area and its vicinity, as illustrated in Figure 2, with each site equipped with a field recorder. The selected monitoring sites encompassed both areas affected by construction noise and pristine ecological zones substantially unaffected by construction activities. Zones E and W are situated within noise‐affected areas, whereas zones WS and WN are located away from noise sources, corresponding to natural areas. The recorders used were waterproof and solar‐powered. They were programmed to record for 1 min every 5 min in a single‐channel, 16‐bit AAC format with a sampling rate of 44,100 Hz. The study period spanned from June 2023 to February 2025, during which approximately 15 TB of continuous audio samples were collected.

**FIGURE 3:**
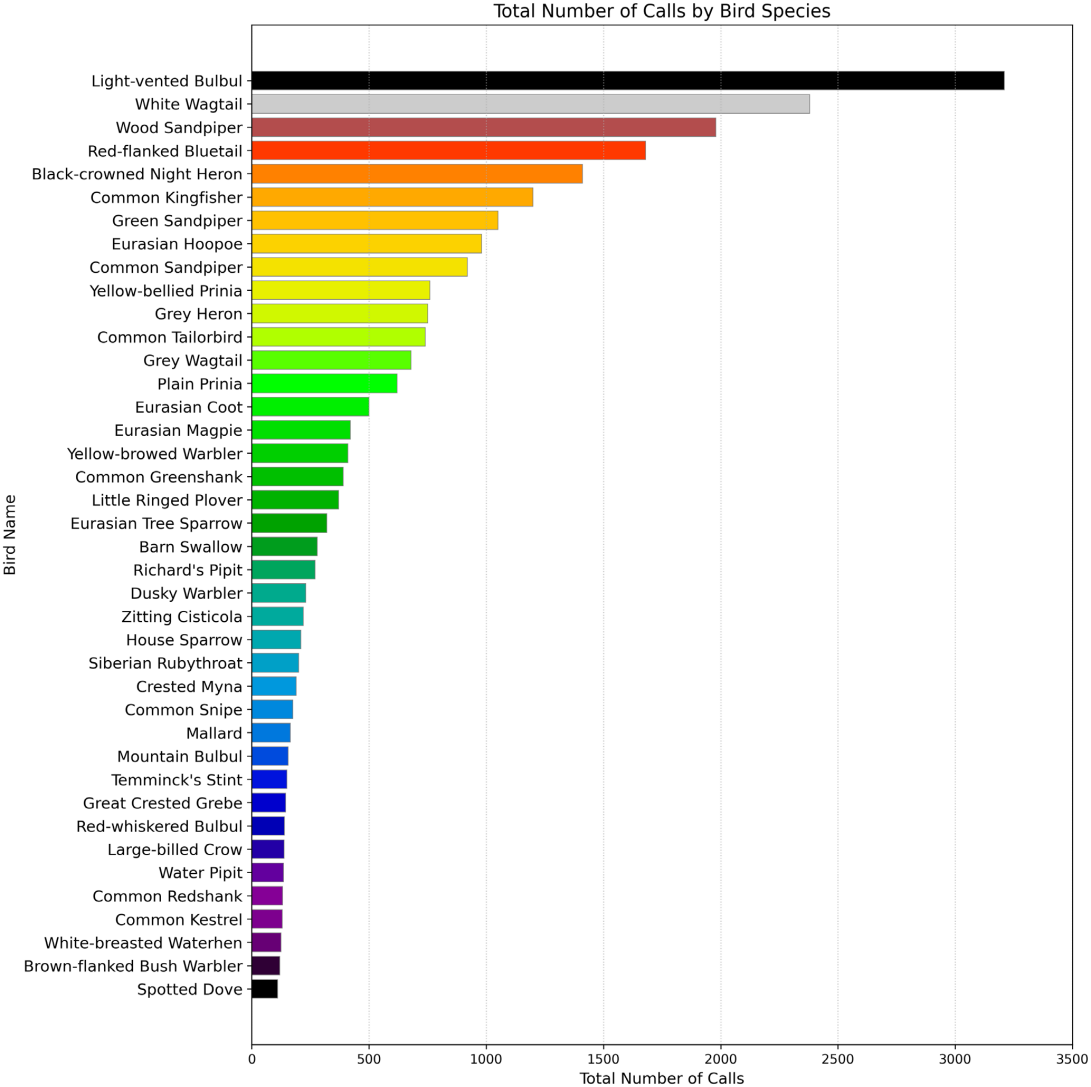
Top 40 bird species with the highest vocalization activity in the Huangmao Sea cross‐sea channel area.

The dataset used for training and evaluating the separation model was primarily downloaded from the Xeno‐Canto website. We initially screened for recordings with minimal background noise that were labeled as containing only a single bird species, with most original audio files ranging in duration from 2 s to 5 min. Regarding species selection, our preliminary survey indicated that a total of 179 bird species were recorded in the recording area, with data sourced from the China Bird Watching Record Center. The top 40 bird species that make the most vocalizations are shown in Figure 3. To control the complexity of model parameter tuning, we selected 20 species from the top 40 bird species with the most frequent vocal activity in this region (including the common nocturnal Black‐crowned Night Heron and diurnal White Wagtail required for this study). Additionally, 10 species were randomly sampled from the remaining 139 bird species, ultimately resulting in a total of 30 bird species selected to construct the dataset. The selected birds included the Black‐crowned Night Heron and White Wagtail required for subsequent experiments; the reason for their selection is that these two species are common and typical nocturnal and diurnal birds in the Huangmaohai region, respectively. All selected audio files were uniformly standardized to mono WAV format. Because the dominant frequencies of these 30 bird species are mostly below 8 kHz, the audio sampling rate was uniformly resampled to 16 kHz to reduce computational load while preserving the vast majority of the frequency information.

Because audio files on the Xeno‐Canto platform are user‐uploaded and annotated, they inevitably contain issues such as mislabeling, as well as prolonged silent segments or high‐intensity background noise within longer recordings. To optimize the performance of subsequent bird song separation and recognition, we preprocessed the filtered audio data. First, the original long audio files were standardized through cropping: all audio files exceeding 16 s were truncated to 16 s and subsequently randomly cropped into two independent 4‐s segments; files with durations between 4 and 8 s were randomly cropped into a single 4‐s segment; and original audio segments shorter than 4 s were discarded. Subsequently, the generated 4‐s audio segments underwent further screening. Experienced avian acoustic researchers manually reviewed the spectrograms and waveforms, combined with auditory verification, to discard low‐quality segments. Ultimately, these 4‐s audio clips were divided into training, validation, and test sets before mixing to ensure that audio clips from the same recording would not appear repeatedly in different data partitions.

In this study, we employed a clip‐based counting method. Specifically, drawing on the concept proposed by Navine et al. (2024), we introduced “call density” as the core evaluation metric, defining it as the proportion of clips containing the target species' vocalizations relative to all clips within a given sampling period. This metric is consistent with the vocal activity rate widely used in passive acoustic monitoring (Jahn et al. 2017). Existing research has well established that this clip‐based proportion of positive detections is significantly and positively correlated with manually verified actual vocalization frequencies (Cole et al. 2022). Therefore, utilizing call density (or VAR) not only effectively circumvents statistical biases caused by cross‐segment truncation but also serves as a robust ecological proxy for assessing avian activity intensity and population dynamics. The audio mixing process involved randomly selecting audio data from *N* (where *N* = 2, 3, 4) species out of the 30 selected bird species. The mixing was performed according to the following formula:

(1) $$\text{Mixed}\left[n\right]=\sum_{i=1}^{N}\;w_{i}\cdot {\text{data}}_{i}\left[n\right]$$

where mixed[*n*] represents the value of the mixed audio signal at time point *n*. *N* represents the number of sound sources, and data_*i*_ [*n*] denotes the value of the audio signal of the *i* sound source at time point *n*. $w_{i}=10^{\mathrm{SN}R_{i}/20}$ represents the weighting coefficient of the *i* sound source. The SNR (Signal‐to‐Noise Ratio) is used to control the relative intensity of each sound source in the mixed audio, and its value is set as a random number within the range of −5 to +5, depending on the number of sound sources in the mixture.

To avoid clipping caused by excessive amplitude of the sound sources, normalization is performed before mixing. The normalization process is conducted according to the following formulas:

(2) $$\begin{array}{c} \text{Scale}=\frac{0.9}{\max\left(\left|\text{mixed}\right|,\left|{\text{data}}_{1}\right|,\left|{\text{data}}_{2}\right|,\ldots ,\left|{\text{data}}_{N}\right|\right)} \end{array}$$

(3) $$\begin{array}{c} {\text{Mixed}}_{\mathrm{norm}}\left[n\right]=\text{mixed}\left[n\right]\cdot \text{scale} \end{array}$$

(4) $$\begin{array}{c} {\text{Data}}_{i,\mathrm{norm}}\left[n\right]={\text{data}}_{i}\left[n\right]\cdot \text{scale} \end{array}$$

where $\max\left(|\text{mixed}|,|{\text{data}}_{1}|,|{\text{data}}_{2}|,\ldots ,|{\text{data}}_{N}|\right)$ represents the maximum absolute amplitude among the mixed audio and all individual sound sources. To ensure the safety of the mixed audio and preserve sound quality without distortion, the scaling factor scale is required to constrain the amplitude of both the mixed audio and each individual source to not exceed 0.9.

To further simulate the complex acoustic conditions of the field, we incorporated environmental noise augmentation into the dataset construction process. We extracted representative background noise segments, including wind, rain, and insect noises, from the 15 TB of field recordings collected in the Huangmaohai region. These noise profiles were linearly added to the multi‐species mixed audio, with the signal‐to‐noise ratio (SNR) between the bird vocalizations and the background noise randomly varying between −5 and 10 dB. This noise‐augmented dataset ensures that the model can learn to disentangle target signals from overlapping biotic sounds and abiotic environmental interference. Ultimately, we mixed 50,000 audio samples. The ratio of mixed audio samples in the training set, validation set, and test set was 7:2:1, which means 35,000 mixed audio segments in the training set, 10,000 in the validation set, and 5000 in the test set.

### 2.2 Model Architecture for Bird Song Separation

**FIGURE 4:**
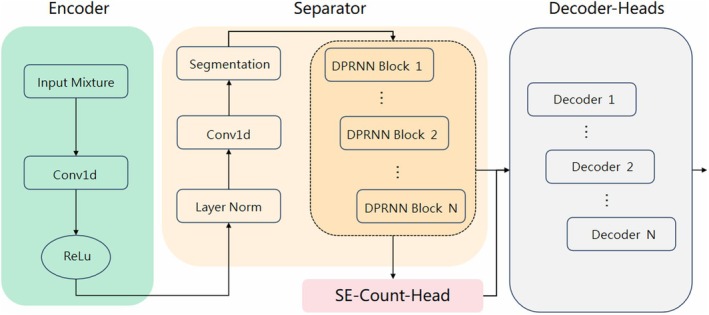
Overall architecture of the proposed model. It primarily consists of an encoder, a separator, a counter, and a selector decoder.

The end‐to‐end bird song separation network used in this study is built upon the sound source separation architecture proposed by Zhu et al. (2021). It primarily consists of an encoder, a separator, a counter, and a selector decoder, as illustrated in Figure 4. Briefly, the encoder first transforms the input mixed audio into a high‐dimensional feature representation, which then undergoes sound source separation via the separator. Subsequently, the counter estimates the number of mixed sound sources and selects an appropriate decoder to decode and reconstruct a waveform for each sound source (a 4‐s audio clip in this paper). Only parameter modifications were made to the encoder, separator, and decoder components used herein. To maintain focus on the core contributions of this paper, detailed descriptions and parameter settings for these components are provided in Appendix A.

The function of the counter is to estimate the number of sources within the audio and subsequently feed this information into the corresponding decoder to reconstruct the audio waveform. This component is designed as a neural network, essentially performing a multi‐class classification task aimed at predicting the number of sound sources in the mixed audio. Specifically, the classes of this classifier are integers representing the number of sound sources, and its output is an integer between 1 and *N* (where the maximum number of sound sources is set to *N* = 4 in this study), serving as a categorical prediction of the source count in the audio. In conventional baseline models for sound source separation (e.g., human speech separation), the counter is typically implemented as a simple neural network using a single‐layer 1 × 1 convolution and global pooling. This foundational structure linearly projects the aggregated feature vectors into a set of logit values corresponding to candidate source counts, selecting the most probable number of sources via an argmax operation. This traditional counter typically relies on the energy distribution and time‐frequency overlap patterns of the speech signals (Zhu et al. 2021). Because these patterns are relatively regular in speech mixtures, the model can capture sufficient information through global pooling alone. Therefore, this traditional method demonstrates good performance in handling speech separation tasks (Stöter et al. 2018; Cosentino et al. 2020). However, bird songs typically possess richer mid‐to‐high frequency components, more complex temporal patterns, and diverse acoustic characteristics (Catchpole and Slater 1995). Merely using a 1 × 1 convolution and adaptive average pooling is insufficient to capture the high‐frequency information and diverse acoustic properties of bird vocalizations. Consequently, this simple counter architecture performs poorly when applied to bird songs, exhibiting even greater instability particularly in scenarios involving multiple bird mixtures or complex background noise. Therefore, it is necessary to design a stable counter module adapted to the signal characteristics of bird songs, which are rich in mid‐to‐high frequency information.

**FIGURE 5:**
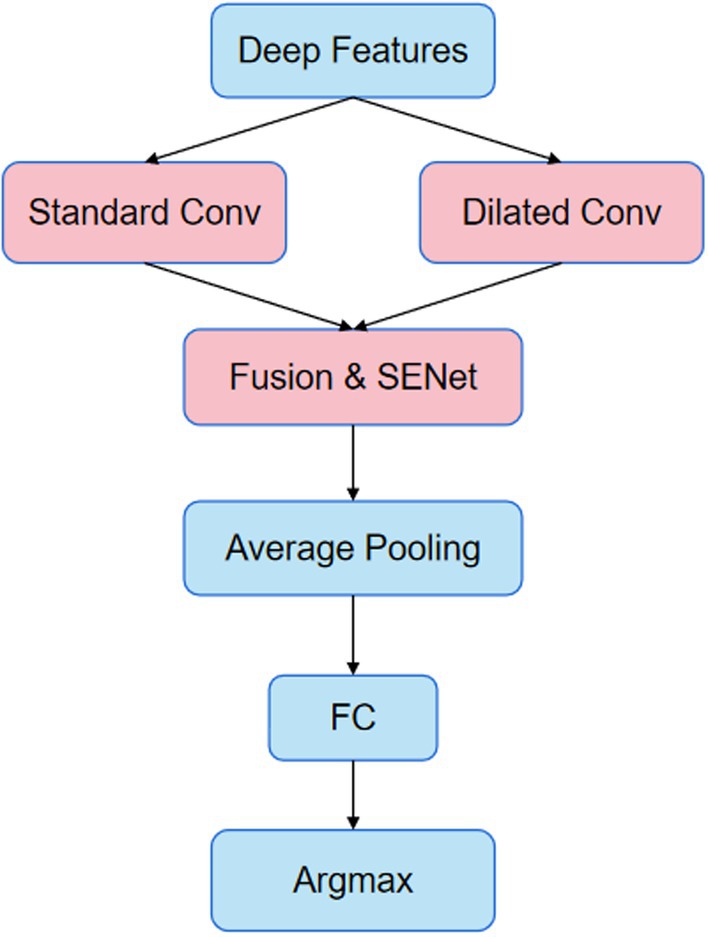
Enhanced counter module architecture, with pink areas indicating improved components, including void convolution and the addition of a SENet module.

In this study, the design of the counter module was improved, with the specific architecture illustrated in Figure 5. The input to the counter module is derived from the deep features at the end of the separation stage. These features are processed to extract both local and global features through different convolutional methods. Specifically, the Standard Conv branch utilizes a 3*3 convolution to extract local features (high‐frequency details) of the bird songs, while the Dilated Conv branch employs dilated convolutions to capture broader global features. The outputs of these two branches are concatenated along the channel dimension to fuse local and global features, thereby capturing a wider frequency range that corresponds to both the high‐frequency and temporal details of bird vocalizations. Furthermore, an SENet module is incorporated to adaptively recalibrate channel attention, enhancing the sensitivity of features related to the number of sound sources (Hu et al. 2018). This mechanism ensures the stable performance of the counter in bird sound analysis.

### 2.3 Model Experiment Setup

In this study, the maximum number of simultaneous vocal sources handled by the model was set to 4. This setting is based on two considerations: first, in complex real‐world field soundscapes, instances where more than four target bird species severely overlap within the same 4‐s audio segment are rare; second, setting a reasonable upper limit helps balance computational efficiency with memory consumption, making the model easier to deploy on resource‐constrained devices. Although the current model imposes this limit, the architecture is scalable; if an application scenario requires processing more complex “avian choruses,” it only requires adjusting the output head and retraining using a dataset containing a higher number of mixed sources. The model learns to separate individual sound sources to effectively represent the mixed soundscape, but it relies on independent Sigmoid masks and imposes no hard mathematical constraints to force the sum of the separated output signals to perfectly equal the original input mixture. Furthermore, the number of dual‐path modules in the model was set to 6, with an initial learning rate of 0.001. The model utilized the Adam optimizer and was trained for 100 epochs, with the learning rate decaying by a factor of 0.98 every two epochs. Because the separated signals might be significantly louder or quieter than the input signals, we employed the Scale‐Invariant Signal‐to‐Noise Ratio (SI‐SNR) as the loss function to disregard irrelevant volume fluctuations, thereby enabling the network to focus on the frequency patterns and timbre essential for robust bird classification. SI‐SNR is a commonly used loss metric in speech separation and a crucial indicator for measuring the similarity between the estimated signal and the clean reference. The unit of SI‐SNR is decibels (dB); a higher score indicates greater similarity between the estimated signal and the clean reference signal, reflecting better separation performance. For a specific sound source, let *s* denote the clean ground‐truth waveform and $\hat{s}$ denote the estimated waveform. The SI−SNR is defined as:

(5) $$\begin{array}{c} \mathrm{SI}-\mathrm{SNR}\left(\hat{s},s\right)=10\log_{10}\left(\frac{{\left\|\mathrm{\alpha s}\right\|}^{2}}{{\left\|\hat{s}-\mathrm{\alpha s}\right\|}^{2}}\right) \end{array}$$

where $\alpha =\frac{s^{T}\hat{s}}{{\left\|s\right\|}^{2}}$ is a scaling factor used to align the amplitude of the target signal with the estimated signal, ensuring that this metric depends only on the signal shape rather than the absolute energy.

## 3 Bird Song Separation Performance and Recognition Results Before and After Separation

After pretraining the bird song separation model, its separation performance and recognition accuracy were validated using the previously proportionally partitioned test set.

### 3.1 Accuracy Results of Estimated Source Quantity by Counter Module

This section aims to explicitly evaluate the accuracy of the counter module in predicting the correct number of sound sources within mixed audio. Table 1 presents the prediction accuracy of the baseline counter versus the improved counter under different levels of sound source mixing complexity. It is evident that, owing to the complex mid‐to‐high frequency temporal characteristics of bird song signals, the baseline model relying solely on simple convolutions struggles with multi‐bird estimation, yielding an accuracy of only 77.6% in the 3‐source scenario. In contrast, the enhanced counter module proposed in this study effectively overcomes the limitations of the single structure by introducing dilated convolutions to expand the receptive field and incorporating SENet for adaptive feature calibration. Consequently, the accuracy exceeded 96% across all mixing scenarios. Specifically, in the 3‐source and 4‐source scenarios, the accuracy improved by 18.8% and 15.2%, respectively, compared to the baseline, thereby providing highly reliable priors for the subsequent dynamic separation decoding.

**TABLE 1:** Accuracy (percentage %) of the counter predicting the number of correct sources.

Count‐head	Count‐head‐bird	Se‐Count‐head (our)
True number of sources		
2	87.1	**99.3**
3	77.6	**96.4**
4	81.9	**97.1**

*Note:* The value in bold is the highest value among the comparison methods used for each line.

### 3.2 Comparison of Separation Model Performance

For bird song separation detection, a custom test set was selected to evaluate separation performance and compare the capabilities of different baseline models. Specific results are shown in Table 2.

**TABLE 2:** Comparison of SI‐SNR performance (dB) of bird song separation model for separating mixtures of different sound sources.

Model	BACPPNet	Mixit	Our
True number of sources			
2	**17.5**	16.2	17.4
3	14.4	14.8	**15.6**
4	*	13.1	**13.5**

*Note:* The bolded values are the highest values among the comparison methods in each row.

* Indicates that the model cannot test the values.

As shown in the table, the BACPPNet model achieves the best performance in two‐source bird song separation tasks; however, constrained by its fixed network architecture, it cannot be extended to 4‐source separation tasks. As an unsupervised separation model, the Mixit model achieves performance comparable to that of supervised separation models. Meanwhile, the model utilized in this study demonstrates strong competitiveness among various bird song separation methods, exhibiting particularly good results in four‐bird mixture scenarios. Furthermore, unlike these baseline models that output a fixed number of channels, our model adopts a dynamic “count‐and‐separate” mechanism. This represents a critical advantage in ecological monitoring scenarios. The counting process provides information regarding the number of mixed bird songs in field audio recordings, and when combined with dynamic separation, it enables the dynamic extraction of individual bird song samples.

### 3.3 Analysis of the Impact of Separation on Bird Song Recognition

To evaluate how different sound source separation models affect downstream classification performance in complex soundscapes, we conducted a comparative analysis. Using the constructed high‐noise test dataset, we evaluated our proposed model alongside two baseline models employing the following metrics commonly used in classification tasks: class‐weighted mean average precision (CMAP), label‐weighted label‐ranking average precision (lwlrap), area under the ROC curve (AUC), and Top‐1 precision. For each model, we tested three input strategies: feeding only the raw mixed signal (Mix), feeding only the separated channels (Separation), and feeding both the mixed signal and the separated channels simultaneously (Mix + Separation), a strategy referenced from Denton et al. (2022). The bird song recognition model utilized was ECAPA‐TDNN (Hu et al. 2025), which has recently demonstrated excellent performance in field bird song recognition and was similarly applied in research within the Huangmaohai Sea‐crossing Channel region. The evaluation results are presented in Table 3.

**TABLE 3:** Classification performance of different separation models and input strategies.

Model	Strategy	CMAP	lwlrap	AUC	Top‐1
BACPPNet	Mix	0.525	0.568	0.723	0.640
	Separation	0.513	0.552	0.717	0.623
	Mix + Separation	**0.529**	**0.579**	**0.734**	**0.657**
Mixit	Mix	0.525	0.568	0.723	0.640
	Separation	0.529	0.576	0.737	0.645
	Mix + Separation	**0.553**	**0.589**	**0.749**	**0.677**
Our	Mix	0.525	0.568	0.723	0.640
	Separation	0.535	0.579	0.748	0.643
	Mix + Separation	**0.558**	**0.594**	**0.752**	**0.676**

*Note:* The bold value is the highest value among the comparison methods in the same group.

As can be seen from the results in Table 3, the “Mix + Separation” strategy consistently yielded the best performance across all three models, indicating that retaining the original mixed signal alongside the separated channels can provide the classifier with isolated target features and the necessary global context. Secondly, relying solely on the “Separation” (separation‐only) strategy exposed the limitations of certain baseline models. Notably, for BACPPNet, using only the separated channels actually degraded the classification performance compared to the original mixed signal (e.g., AUC dropped from 0.723 to 0.717). This suggests that although the model separated the audio, it might have filtered out critical acoustic features or introduced detrimental artifacts that adversely affected the classifier (Denton et al. 2022). Mixit exhibited moderate improvements under the “Separation‐only” setting. Our proposed model demonstrated results comparable to Mixit, and compared to the baseline models, it achieved the highest overall performance under the combined “Mix + Separation” strategy (CMAP: 0.558, lwlrap: 0.594, AUC: 0.752, Top‐1: 0.676). This validates the effectiveness of our dynamic counting and separation framework in disentangling overlapping vocalizations in complex field environments, while successfully preserving the high‐fidelity acoustic characteristics required for accurate downstream species recognition.

## 4 Analysis of the Impact of Separation on Bird Vocalization Monitoring Results

Based on the impact of the aforementioned separation on downstream classification performance, we used field bird call data collected from monitoring sites and adopted the effective Mix + Separation strategy to analyze the impact of separation on bird vocalization monitoring results. Concurrently, to ensure the ecological validity of the automated monitoring results, we implemented a stratified random sampling procedure to verify the precision and recall before and after separation, thereby addressing potential biases in machine learning recall. We conducted stratified random sampling from the raw audio across different temporal strata (e.g., different hours of the day) to extract a subset of 4‐s audio segments (comprising 30% of the data). These samples were subsequently reviewed by expert ornithologists to ensure greater accuracy. The pre‐trained ECAPA‐TDNN classifier applied a confidence threshold of 0.7, which is based on the approach taken by Kahl et al. in bird call recognition (Kahl et al. 2021). We reported all VAR results as proportional measurements. Furthermore, we introduced a specific metric: the valid increment ratio (VIR), the values of which were all manually verified by experienced ornithologists. The VIR is defined as the number of newly added true positives post‐separation divided by the total number of newly added detections generated by the system post‐separation. This metric specifically calculates the precision for detections that were missed in the raw analysis but “newly generated” by the separation model; therefore, it is exclusively applicable to post‐separation results.

The bird species selected for the experiment are all common in the Huangmaohai Sea‐crossing Channel region. For the study of nocturnal birds, the Black‐crowned Night Heron was used as an example. The Black‐crowned Night Heron is a medium‐sized waterbird of the order Ciconiiformes and family Ardeidae, holding representative significance among Ardeidae species. It is a typical nocturnal bird that usually engages in foraging activities at night (Maccarone and Hamilton 2014; McNeil et al. 1993). Its primary vocalization frequency typically ranges between 1 and 2 kHz. For diurnal birds, we selected the White Wagtail, which is also common in the Huangmaohai Sea‐crossing Channel region. The White Wagtail belongs to the family Motacillidae and the genus Motacilla. It primarily inhabits environments such as watersides and wetlands, with a vocal frequency range between 3 and 7 kHz (Badyaev et al. 1996; Shirazinejad et al. 2019).

### 4.1 Diurnal and Nocturnal Vocal Activity Rhythms in Birds

For the analysis of the circadian vocal activity rhythms of nocturnal and diurnal birds, we conducted experiments using data from a randomly selected month at monitoring site E1, which is located in a noise‐affected area. We first validated the recognition precision and recall for both species before and after separation using this month's data. The validation was conducted by extracting a stratified random sample of 30% of the raw data (approximately 10 days) from that month. The validation results are presented in Table 4. As shown in the table, following sound source separation, the recall improved for both species compared to the raw unseparated data: from 78.2% to 83.4% for the Black‐crowned Night Heron, and from 79.1% to 84.3% for the White Wagtail. This indicates that separation can effectively disentangle target bird songs from masking noise or non‐avian species, thereby improving recall. Concurrently, precision also experienced consistent growth (rising to 79.6% and 79.8%, respectively), which further demonstrates that the separation process effectively suppressed noise interference.

**TABLE 4:** Validation results of random sampling of Black‐crowned Night Heron and White Wagtail data within the selected month.

Species	Strategy	Precision	Recall	Valid increment ratio
Black‐crowned Night Heron	Raw	0.773	0.782	—
	Separated	**0.796**	**0.834**	**0.896**
White Wagtail	Raw	0.769	0.791	—
	Separated	**0.798**	**0.843**	**0.875**

*Note:* The bold value is the highest value among the comparison methods in the same group.

Furthermore, we evaluated the valid increment ratio (VIR), which reached 89.6% for the Black‐crowned Night Heron and 87.5% for the White Wagtail. This indicates that the vast majority of the additional audio segments recovered by the separation model are valid, high‐fidelity target vocalizations, rather than false positive artifacts.

**FIGURE 6:**
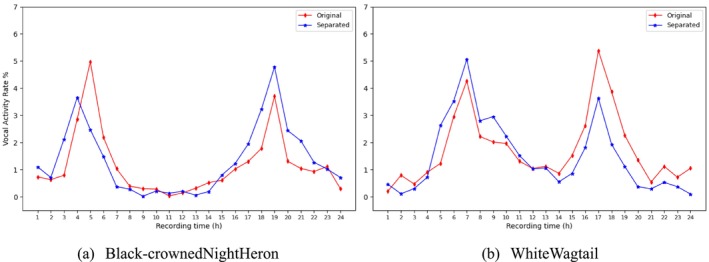
Comparison of average vocal activity before and after separation of Black‐crowned Night Heron and White Wagtail over 1 month.

Based on the aforementioned validation data, the results obtained by separating the field monitoring data for this month are illustrated in Figure 6 for the Black‐crowned Night Heron (a) and the White Wagtail (b). The red line represents the raw automated recognition results, while the blue line represents the recognition results following separation processing.

Observing the curves reveals that the recognition statistics derived from the separated data effectively mitigate the influence of non‐target noise. Specifically, for the nocturnal Black‐crowned Night Heron (Figure 6a), the raw monitoring data during the activity peaks at dawn (04:00–05:00) and dusk (19:00) were significantly underestimated. This indicates that in environments characterized by overlapping sound sources or dawn choruses, target bird songs are easily masked, leading to missed detections (false negatives). The separation algorithm successfully extracted faint Black‐crowned Night Heron signals from the mixed background, bridging the gap of underestimation in the raw detections and authentically restoring its characteristic pattern of vocalizing predominantly at night. Furthermore, for the diurnal White Wagtail (Figure 6b), the raw data exhibited an abnormal false peak during the evening hours (around 17:00). Upon analysis, this was attributed to environmental noise, such as insect calls overlapping with the White Wagtail's frequency band, which resulted in misclassifications (false positives). The separation process successfully suppressed this noise‐induced false peak, returning the activity curve to a normal diurnal rhythm. This aligns with the findings of Ramesh et al., which suggest that diurnal birds exhibit higher activity during the dawn chorus compared to dusk due to their foraging behaviors (Ramesh et al. 2025). In summary, the separated vocal activity curves (blue lines) effectively correct systematic biases in the raw data, significantly enhancing the authenticity and ecological explanatory power of the monitoring results.

### 4.2 Effects of Climate Factors on Vocal Activity Rates

To investigate the effects of environmental factors (temperature, precipitation, wind speed) on the vocal activity rate (VAR) of the two target species, the Black‐crowned Night Heron and the White Wagtail, bird sound data spanning 4 months (October 2023–January 2024) were utilized. These data were collected from monitoring site W4, which is situated in a noise‐affected area. We constructed Generalized Linear Mixed Models (GLMMs). Statistical analysis and data visualization were performed in a Python (3.x) environment, implemented using the scikit‐learn and SciPy libraries. Meteorological data (temperature, rainfall, and wind force) for Jinwan District, Zhuhai, were obtained from the “Tianqi24” website (https://www.tianqi24.com), with the weather station located approximately 10 km from the study area. Considering the potential non‐linear response of avian vocalization to climatic variables, we introduced a third‐order polynomial regression within the GLMM framework. The VAR derived from different sources (raw recognition, manual verification, and separated data) served as the response variable, while environmental factors served as fixed effects. To ensure the reliability of the conclusions, we calculated 95% confidence intervals (CIs) for the predicted means based on the model parameter covariance matrix. Model goodness‐of‐fit was evaluated via residual analysis and the coefficient of determination (*R* ^2^).

Similarly, we first conducted stratified random sampling on the selected data to validate the recognition precision and recall for both species. The validation results are presented in Table 5. As can be seen from the data in the table, both the recall and precision for the two experimental bird species improved following separation. Concurrently, the valid increment ratio (VIR) for both birds achieved favorable results (85.7% and 84.6%, respectively). This indicates that the separation process can effectively suppress the influence of non‐avian noises, and that the vast majority of the additional audio segments recovered by the separation model are valid.

**TABLE 5:** Validation results of random sampling of Black‐crowned Night Heron and White Wagtail data from the four selected months.

Species	Strategy	Precision	Recall	Valid increment ratio
Black‐crowned Night Heron	Raw	0.761	0.776	—
	Separated	**0.789**	**0.813**	**0.857**
White Wagtail	Raw	0.758	0.782	—
	Separated	**0.784**	**0.837**	**0.846**

*Note:* The bold value is the highest value among the comparison methods in the same group.

**FIGURE 7:**
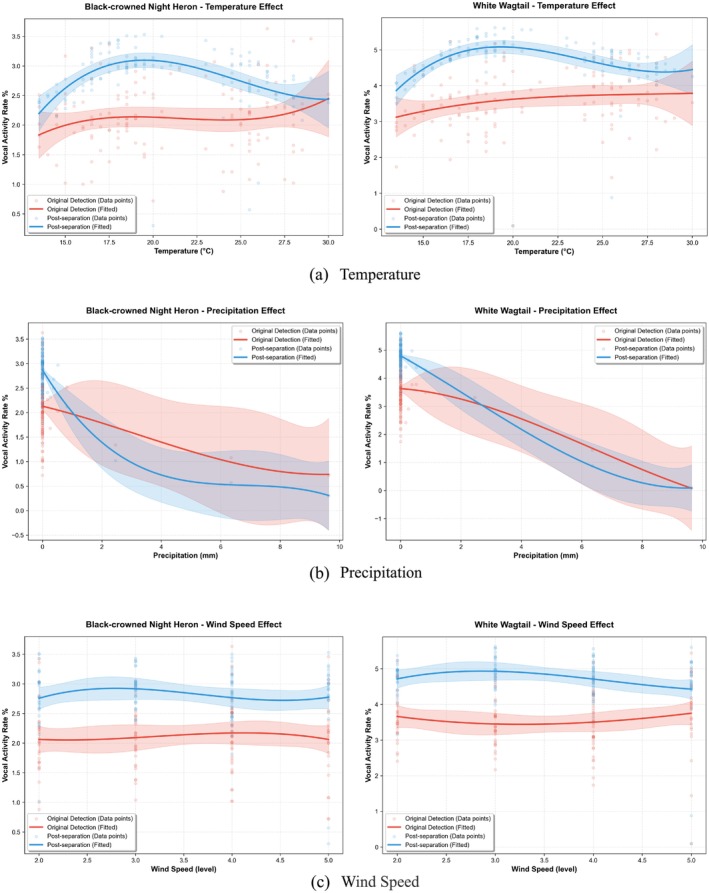
Generalized linear mixture model (GLMM) fitting results of the effects of environmental factors temperature (a), precipitation (b), and wind speed (c) on the vocal activity rate (VAR) of Black‐crowned Night Heron (left) and White Wagtail (right).

Based on the validation results from random sampling, we plotted Generalized Linear Mixed Models (GLMMs) of avian vocal activity rates before and after the separation of bird song data from October 2023 to January 2024, as shown in Figure 7.

This figure shows the relationship between the vocal activity rates of the Black‐crowned Night Heron and the White Wagtail and temperature, precipitation, and wind speed. The red curve represents the raw, unprocessed recognized vocal activity rate, derived from data obtained by the recognizer without separation, while the blue curve represents the vocal activity rate data obtained by the recognizer post‐separation. The results indicate that regarding the influence of environmental temperature, the raw recognition data (red curve) exhibited a flat linear trend with wide confidence intervals for both the Black‐crowned Night Heron (Figure 7a left) and the White Wagtail (Figure 7a right), which was statistically insignificant. However, the separated data (blue curve) revealed a typical non‐linear response that was previously masked, wherein vocal activity reaches its peak within an optimal temperature range and significantly decreases under low or high‐temperature stress.

Secondly, concerning the precipitation factor (Figure 7b), although both sets of data indicated that precipitation has an inhibitory effect on vocal activity, the post‐separation data demonstrated a more precise response threshold. Compared to the underestimated raw detection trend, the separated blue curve exhibits a steeper decay slope, indicating that the birds' sensitivity to rainfall is actually higher than what the raw monitoring suggested; the separation technology successfully corrected the detection bias caused by the masking effect of rain noise. As for the impact of wind speed (Figure 7c), it did not exhibit a significant statistical correlation in any of the models.

Thus, it is evident that the integration of sound source separation technology mitigates the “detection constraint” issue caused by complex acoustic environments. By effectively disentangling overlapping signals, the accuracy and sensitivity of avian vocal activity analysis are improved, which enables linear models to capture the true, high‐fidelity relationships between climatic variables and avian vocal behaviors, ultimately enhancing the statistical power and ecological explanatory power of the study.

### 4.3 Seasonal Patterns and Their Impact on Vocal Activities

Avian vocal behaviors exhibit certain seasonal patterns and are simultaneously influenced by multiple factors (Hao et al. 2025). During the breeding season, birds typically increase their vocalization frequency and intensity significantly to attract mates, mark territories, or deter competitors; this behavior is particularly pronounced in spring and summer (Catchpole and Slater 2003). Conversely, during the non‐breeding season, vocalizations may decrease to conserve energy (Odom et al. 2016).

Based on vocalization data from four distribution sites (E, W, WN, WS), we conducted a 1‐year data analysis for the Black‐crowned Night Heron and the White Wagtail, respectively, with the recording period spanning from June 2023 to May 2024. Because sites E and W are both located within noise‐affected areas, we categorized them as noise zones during validation; similarly, WN and WS were categorized as natural zones. First, we conducted stratified random sampling on the data. The sampling method involved randomly drawing samples (a 30% sample) from each month at each site across the 1‐year timeline to validate the recognition precision and recall for the Black‐crowned Night Heron and the White Wagtail before and after separation. The validation results are shown in Table 6.

**TABLE 6:** Validation results of random sampling of Black‐crowned Night Heron and White Wagtail data from the selected 1‐year period.

Species	Points	Strategy	Precision	Recall	VIR
Black‐crowned Night Heron	Noise areas	Raw	0.752	0.769	—
		Separated	**0.773**	**0.781**	**0.837**
	Natural areas	Raw	0.768	0.789	—
		Separated	**0.775**	**0.797**	0.791
White Wagtail	Noise areas	Raw	0.763	0.766	—
		Separated	**0.791**	**0.787**	**0.876**
	Natural areas	Raw	0.776	0.786	—
		Separated	**0.779**	**0.794**	0.828

*Note:* The bold value is the highest value among the comparison methods in the same group.

As can be seen from the sampling validation results in Table 6, the sound source separation process improved precision and recall to varying degrees across all monitoring sites. Crucially, the magnitude of this improvement largely depends on the specific acoustic environment. In noise‐affected areas, raw baseline recognition performance was significantly suppressed due to the masking effect of environmental sounds. Following the application of sound source separation, both precision and recall for the two species achieved a notable improvement (e.g., the precision of the White Wagtail in noisy areas increased from 0.763 to 0.791). In contrast, in natural areas with minimal background noise, the separation process provided a slight yet positive optimization to the already robust baseline data. Furthermore, the valid increment ratio (VIR) was higher in noise‐affected areas compared to natural areas (reaching 0.837 for the Black‐crowned Night Heron and 0.876 for the White Wagtail). This indicates that in disturbed environments, the vast majority of the acoustic events newly recovered by the model are valid bird vocalizations. This validation demonstrates that the separation framework effectively mitigates spatial detection biases caused by overlapping noise, thereby leveling the detection capabilities of recording devices across diverse soundscapes.

**FIGURE 8:**
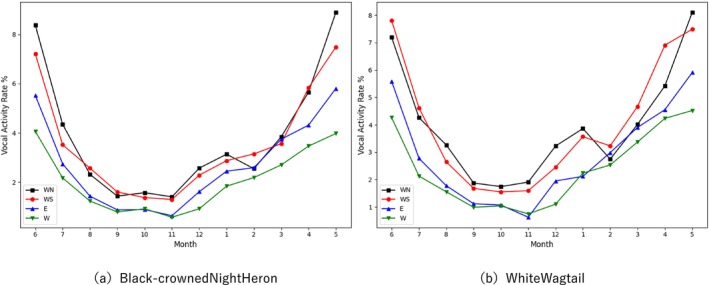
Variations in vocal activity of the Black‐crowned Night Heron (a) and the White Wagtail (b) at different monitoring sites from June 2023 to May 2024.

Figure 8 illustrates the changes in the vocal activity rates of the Black‐crowned Night Heron and the White Wagtail across different site locations from June 2023 to May 2024, based on the separated data. The blue, green, black, and red lines represent the vocalization data from the four distribution sites (E, W, WN, and WS, respectively), with the left panel displaying the Black‐crowned Night Heron and the right panel displaying the White Wagtail.

From a temporal perspective, the vocal activity of the Black‐crowned Night Heron exhibits a significant seasonal rhythm. Its primary active period spans from November to June of the following year, and with the onset of the breeding season, the VAR reaches its peak in May (approximately 6%–8%). This also corroborates the driving effect of courtship and territorial behaviors during the breeding season on vocalization. Conversely, during the non‐breeding season (e.g., September–October), vocal activity significantly decreases, with the VAR dropping below 2%. From a spatial perspective, although the seasonal variation trends remain consistent across different monitoring sites, differences exist in the absolute VAR values. Notably, the VAR recorded in noise‐disturbed areas (E, W) is generally lower than that in natural ecological areas (WN, WS). Similarly, Figure 8b demonstrates that the White Wagtail exhibits a seasonal fluctuation pattern highly similar to that of the Black‐crowned Night Heron, peaking during the height of the breeding season (May–June) and bottoming out in autumn. Its spatial distribution differences are equally significant: the curves for the noisy sites (E, W) lie below those of the natural sites (WN, WS) almost year‐round. These results suggest that bird vocalization exhibits seasonal variations, similar to the findings of Puswal et al. in their study of birds in China (Puswal and Liang 2024), and that noise may have a negative impact on detection probability, or may be due to noise reducing bird vocalization activity (McClure et al. 2013). Furthermore, this indicates that although nocturnal and diurnal birds differ starkly in their circadian rhythms (as shown in Figure 6), they exhibit convergence in their long‐term seasonal breeding rhythms.

## 5 Discussion

### 5.1 Effect of Separation Model Algorithms on Estimation of Vocal Activity Rate

In terms of model architecture, this study adopted an end‐to‐end dual‐path network combined with an improved counter module. Unlike previous studies constrained by a fixed number of output channels, our design effectively addresses the “unpredictable source count” issue prevalent in real‐world passive acoustic monitoring (PAM). More importantly, our evaluation using common classification metrics (such as AUC and CMAP) and the “Mix + Separation” strategy reveals a key insight: combining the separated channels with the original mixed audio can provide the classifier with both isolated target features and the necessary global context (Denton et al. 2022). This hybrid approach significantly improves overall classification robustness, demonstrating that sound source separation is an effective front‐end enhancement tool for bioacoustic recognition in noisy, multi‐species environments.

### 5.2 Discussion on the Effects of Separation on Bird Vocalization Activity

#### 5.2.1 Effects of Separation on Vocalization Activity in Diurnal and Nocturnal Birds

By standardizing VAR as a proportional measurement and validating it through rigorous stratified random sampling, this study demonstrates that mixed sound source separation technology plays a crucial role in revealing the true circadian vocalization rhythms of birds. In the raw audio monitoring without separation, the White Wagtail exhibited an abnormal vocal peak during the evening hours (Figure 6b). Studies have shown that, driven by light and social behaviors, the vocal activity of diurnal birds generally reaches its daily peak at dawn, exhibiting a dawn bias (Ramesh et al. 2025). By comparing these results with expert count data, it was found that this false peak was primarily attributed to high‐intensity insect noise (cicada calls) in the evening within the Huangmaohai region, as well as overlapping interference from other bird species active during the same period. These noises were misclassified by the recognition model as the target species, leading to a “false positive” overestimation of the vocal activity rate (VAR), which is a common phenomenon in automated monitoring (Pérez‐Granados 2023). The separation model successfully filtered out these background noises and interferences, eliminating the false evening peak and restoring the typical bimodal pattern of the White Wagtail, which is dominated by dawn vocalizations.

Conversely, for the nocturnal Black‐crowned Night Heron, the raw data significantly underestimated its nocturnal vocal intensity (Figure 6a). This aligns with the “masking effect” in passive acoustic monitoring, where wind noise or chorus phenomena in the nocturnal environment mask the target signals (Barber et al. 2010). The separated data recovered the masked faint signals, rendering its nocturnal activity curve highly consistent with the expert count data and accurately reflecting its active patterns of nocturnal socialization as a nocturnal bird. This result confirms that in complex acoustic environments, directly relying on raw audio for automated monitoring may introduce significant systematic biases due to sound source overlap, whereas sound source separation is an effective means of correcting such temporal allocation biases.

#### 5.2.2 Enhancing the Isolation of Statistical Sensitivity to Environmental Factors

In the analysis of the influence of climatic factors on vocal activity rate (VAR), the introduction of sound source separation technology significantly enhanced the explanatory power of the statistical models. According to the Acoustic Adaptation Hypothesis, environmental conditions (such as temperature and humidity) directly influence avian vocalization strategies by altering the attenuation characteristics of sound waves (Morton 1975; Boncoraglio and Saino 2007). However, in mixed audio, overlapping sound sources act as random error terms, reducing the signal‐to‐noise ratio and statistical power of regression models, thereby making it difficult for the models to capture avian responses to subtle climatic changes. Our Generalized Linear Mixed Models (GLMMs) showed that prior to separation, the effect of temperature on the VAR of both bird species did not reach statistical significance. In contrast, after separation processing, temperature exhibited a significant negative correlation (Figure 7). This shift reveals micro‐ecological responses previously masked by “noisy” data. The “purification” effect of the post‐separation data enabled us to observe the behavioral strategies of the Black‐crowned Night Heron and the White Wagtail, which significantly reduced their vocal activity rates under conditions of high temperature and rainfall. This characteristic aligns with findings on how relevant climatic factors affect avian vocal activity (Pérez‐Granados and Schuchmann 2021b). This behavior serves not only to circumvent unfavorable acoustic transmission conditions but may also represent an energy trade‐off mechanism under heat stress or rainfall disturbance (du Plessis et al. 2012). This finding underscores that failure to address the issue of sound source overlap may lead researchers to underestimate the potential impacts of climate change on avian behavioral patterns.

### 5.3 Seasonal Rhythms of Avian Vocal Activity and Noise Effects

Based on the long‐term separated data, this study reveals significant differences in the temporal and spatial dimensions of avian vocal activity in the Huangmaohai region. As shown in Figure 8, although the Black‐crowned Night Heron (nocturnal) and the White Wagtail (diurnal) differ starkly in their circadian rhythms, they exhibit a high degree of phenological synchrony in their long‐term seasonal variations and display consistent noise response patterns across different soundscape regions.

First, in the temporal dimension, the vocal activity peaks of both target bird species are concentrated between May and June, which highly aligns with the typical breeding cycle of subtropical birds. According to theories related to passive acoustic monitoring (PAM), male songbirds significantly increase their singing frequency during the breeding season for courtship displays and territorial defense; this behavior is strictly regulated by seasonal photoperiods and sex hormone levels (Pérez‐Granados and Schuchmann 2021b; Digby et al. 2014). The separated data accurately captured this “breeding chorus” driven by physiological cycles, demonstrating the sensitivity of this technology in long‐term phenological monitoring.

Second, regarding spatial distribution, the number of bird song detections in strong noise interference zones (sites E and W) was generally significantly lower than that in natural ecological zones (sites WN and WS). This phenomenon may be due to the negative impact of noise on the detection probability, or it may be caused by the “Noise Avoidance Hypothesis.” Previous experimental studies have shown that long‐term human traffic or construction noise can create acoustic barriers, forcing birds that are sensitive to the sound environment to reduce their distribution in the area, or reduce their willingness to vocalize due to communication obstruction (McClure et al. 2013). Noise acts as an “ecological filter,” leading to a decline in bioacoustic abundance in disturbed areas (Francis et al. 2009).

## 6 Summary

This study addresses the challenges of multi‐source overlap and uncertainty in the number of sources faced by Passive Acoustic Monitoring (PAM) in complex field environments. We proposed and verified an improved end‐to‐end bird sound separation and analysis framework, successfully achieving dynamic counting and high‐fidelity separation of mixed bird songs with an unknown number of sources in the wild. Comparative analysis experiments conducted on the separated data yielded several conclusions. First, bird sound separation significantly improved recognition reliability in complex soundscapes. Experimental results indicate that the model effectively deconstructs overlapping acoustic events, significantly increasing the recognition confidence of target species within overlapping segments. Compared to direct recognition of raw audio, the separation process effectively corrected “false positive” misclassifications caused by background noise (such as insect sounds) and interference from non‐target bird songs, thereby ensuring the accuracy of species diversity statistics.

Furthermore, the separation technology restored the true circadian activity rhythms of birds and revealed that acoustic data processed without separation could lead to misinterpretations of avian behavioral patterns; the framework proposed in this study effectively eliminates such systematic biases. Finally, the separated data unveiled previously masked environmental driving mechanisms. Analysis using Generalized Linear Mixed Models (GLMMs) demonstrated that sound source separation significantly enhanced the explanatory power of climatic variables regarding vocal activity rates. The separated data clearly exhibited the non‐linear inhibitory effects of temperature and precipitation on avian vocal activity, validating the ecological hypothesis that birds adjust their vocalization strategies to adapt to environmental stress—a critical correlation that was statistically insignificant in the raw mixed data.

Overall, this study validates the significant application value of sound source separation technology in avian acoustic monitoring, markedly improving the scientific rigor of recognition and analysis in multi‐species chorus scenarios. The analysis framework constructed in this study provides a robust scientific tool for conducting precise biodiversity monitoring and assessment in the Huangmaohai Sea‐crossing Channel and other areas subject to high‐noise interference.

## Author Contributions

**Jie Wang:** formal analysis (equal), investigation (equal), methodology (equal), validation (equal), visualization (equal), writing – original draft (equal), writing – review and editing (equal). **YanChao Lai:** conceptualization (equal), data curation (equal), formal analysis (equal), investigation (equal), methodology (equal), software (equal), writing – original draft (equal). **Xuehan Wang:** methodology (equal), validation (equal), visualization (equal), writing – review and editing (equal). **Jinhui Li:** validation (equal), visualization (equal), writing – review and editing (equal). **Yanqin Wang:** conceptualization (equal), methodology (equal), validation (equal), visualization (equal), writing – review and editing (equal). **Shegang Shao:** supervision (equal), validation (equal), visualization (equal), writing – original draft (equal). **Minmin Yuan:** validation (equal), visualization (equal), writing – review and editing (equal).

## Funding

This work was supported by the National Key Research and Development Program, 2024YFF1307700, the Central basic scientific research fund, 2025‐9021A, the National Natural Science Foundation of China, 62501182, the University‐Enterprise Joint Funding Project of Guangzhou City, 2025A03J3092, 2025A04J4589.

## Conflicts of Interest

The authors declare no conflicts of interest.

## Figures and Tables

**FIGURE A1:**
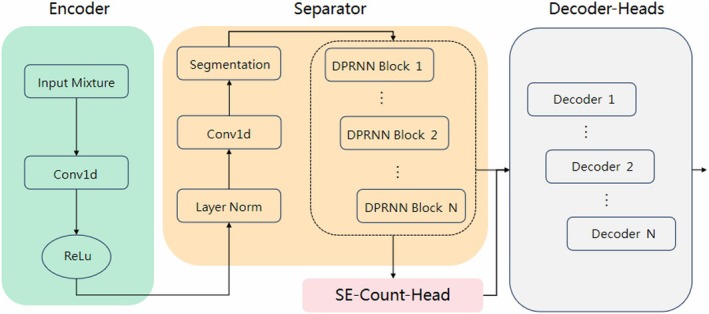
Overall architecture of the proposed model.

**FIGURE A2:**
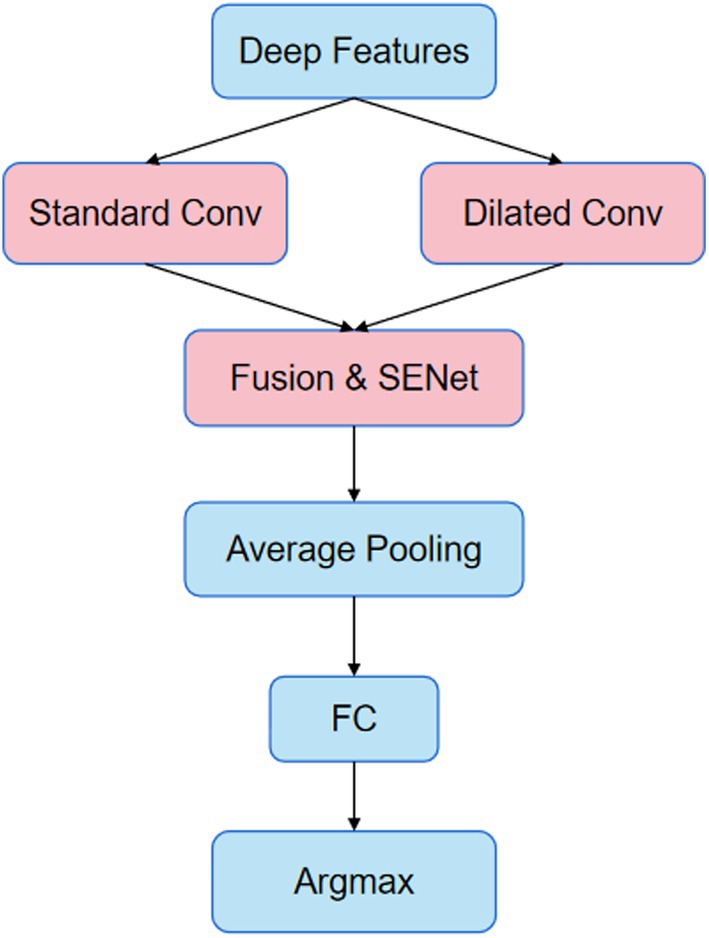
Enhanced counter module architecture, with pink areas indicating improved components, including void convolution and the addition of a SENet module.

**FIGURE A3:**
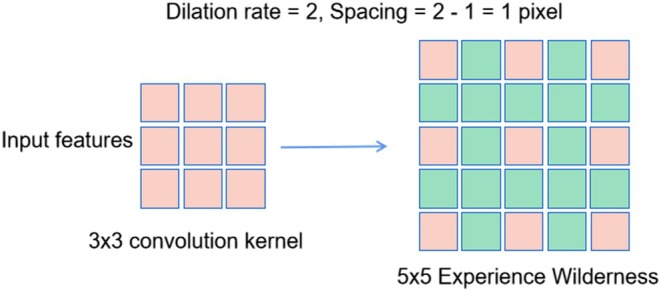
Schematic diagram of 3 × 3 dilated convolution with a dilation rate of 2.

**FIGURE A4:**
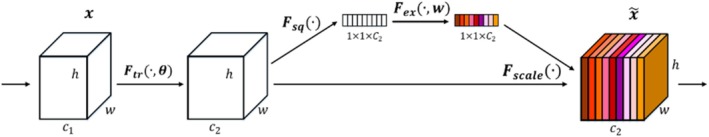
SENet architecture.

## Data Availability

The source code for the proposed supervised multi‐source bird song separation framework is publicly available in the Zenodo repository, DOI: https://doi.org/10.5281/zenodo.19601771. And the model architecture and code are located at this website: https://github.com/txfstarss‐blip/Bird‐Song‐Separation. The data required for the experiment is located at this website: https://drive.google.com/drive/folders/1mCb_‐_89Twa6tCvWJ8YXgZJ9Rl8VQq3‐?usp=sharing.

## Appendix A Model Architecture Used for Birdsong Separation

The end‐to‐end bird call separation network used mainly consists of an encoder, a separator, a counter, and a selector decoder, as shown in Figure A1. In simple terms, the encoder first converts the input mixed audio into a high‐dimensional feature representation. The separator then separates the sound sources. Next, the counter estimates the number of mixed sound sources and selects an appropriate decoder to decode and reconstruct the waveform. Finally, the aliased audio segments are separated into several audio segments containing only one type of bird call.

### Encoder

The encoder uses one‐dimensional convolutional layers to $x\in R^{1\times T}$ transform the time‐domain waveform into a high‐dimensional latent feature representation $w\in R^{N_{\mathrm{enc}}\times L}$. Here, $N_{\mathrm{enc}}$(default 64) represents the number of filters, acts as the learnable frequency band, and L represents the number of time frames, determined by the convolution stride. Unlike the fixed basis of STFT, this learnable representation captures the unique time‐frequency characteristics of bird calls, mathematically serving as a “pseudo‐spectral map” for separation. A ReLU activation function is applied after the convolution to ensure non‐negativity.

### Separator

The splitter employs a dual‐path recurrent neural network (DPRNN) architecture. First, the bottleneck layer projects the encoder features onto a dimension N(set to 128). The segmentation module then divides this sequence into K overlapping blocks of size (set to 100), reshaping the data into a 3D tensor $D\in R^{N\times K\times S}$, where S is the number of blocks. This tensor is processed by stacked DPRNN blocks. Each block contains two stages: (1) intra‐block processing, applying a K bidirectional RNN to the dimension to capture local short‐term dependencies; (2) inter‐block processing, applying a *S* bidirectional RNN to the dimension to model long‐term global dependencies. Finally, the processed tensor is folded back using the Overlapped Addition (OLA) method, the inverse of the block operation, used to reconstruct the processed segmented data back into a continuous time series.

Assume we have a processed block sequence $Y_{s}\in R^{N\times K}$, where s=1 is …,S the block index. Set the hop size to P(typically K/2, i.e., 50% overlap). The reconstructed sequence $O\left[t\right]$ is calculated as follows:

$$O\left[t\right]=\sum_{s=1}^{S}Y_{s}\left[t-\left(s-1\right)P\right]$$

This $Y_{s}\left[t-\left(s-1\right)P\right]$ indicates that the first s block is shifted by positions on the timeline $\left(s-1\right)P$ and then added to the total output. The values in the overlapping areas will be accumulated.

### Enhanced Counter Module

The counter's role is to estimate the number of sources in the audio and then feed it into the corresponding decoder to reconstruct the audio waveform. This part is designed as a neural network, essentially a multi‐classification task. The counter is implemented as a simple neural network using a single‐layer 1 × 1 convolution and global pooling. This structure linearly projects the aggregated feature vector into a set of logit values corresponding to the number of candidate sources and selects the most probable number of sources from these logit values through an argmax operation. In voice separation, the counter typically relies on the energy distribution and time‐frequency overlap patterns of the speech signal. These patterns are relatively regular in voice mixing, and the model can capture sufficient information through global pooling. Therefore, this method shows good performance in handling voice separation tasks, especially when the training data covers diverse voice mixing scenarios (Stöter et al. 2018; Cosentino et al. 2020). However, birdsong typically has richer mid‐to‐high frequency components, more complex timing patterns, and diverse acoustic characteristics (Catchpole and Slater 1995). Simply using 1 × 1 convolution and adaptive average pooling is insufficient to capture the high‐frequency information and diverse acoustic characteristics of birdsong. Therefore, this simple counter architecture is not effective in birdsong, especially when multiple birds are mixed or the background noise is complex, and its performance is even more unstable. Therefore, it is necessary to design a stable counter module to adapt to the characteristics of birdsong, which has rich mid‐to‐high frequency information.

This study improved the design of the counter module, as shown in Figure A2. The input of the counter module comes from the deep features of the separated ends. First, local and global features are extracted through different convolution methods. The Standard Conv branch represents a 3*3 convolution used to extract local features (high‐frequency details) of birdsong, while the Dilated Conv branch represents a dilated convolution to capture a wider range of global features. Figure A3 shows a schematic diagram of a 3 × 3 dilated convolution with a dilation rate of 2. By introducing a hole, the receptive field is expanded without increasing the number of parameters, thereby capturing a wider range of global contextual information. Then, the outputs of the two branches are fused and spliced along the channel dimension to fuse local and global features to capture a wider frequency range, corresponding to capturing the high‐frequency and temporal details of birdsong. Furthermore, the SENet module is added to adaptively recalibrate the channel attention to enhance the sensitivity of sound source quantity‐related features, thereby making the counter perform stably in birdsong.

The SENet module is to allow the network to adaptively recalibrate channel feature responses, enhancing useful features and suppressing irrelevant features. Furthermore (Hu et al. 2018). The specific architecture is shown in Figure A4, and its process consists of three steps: First, the Squeeze operation × performs global average pooling on the input feature map, compressing the spatial dimension into a single value to obtain the channel description vector $F_{\mathrm{sq}}\left(\cdot \right)$. This step effectively captures the global information of each channel. Next, the Excitation operation inputs the channel description vector into two fully connected layers, performing dimensionality reduction and then expansion, followed by ReLU and Sigmoid activation to generate channel weights ranging from 0 to 1 $F_{\mathrm{ex}}\left(\cdot ,w\right)$, where w represents the learnable weight parameters. Finally, the Scale operation multiplies the generated channel weights element‐wise $F_{\text{scale}}\left(\cdot \right)$ with the original feature map × to obtain a weighted feature map $\tilde{x}$, achieving channel‐level feature recalibration. The SENet module helps extract highly discriminative mid‐to‐high frequency features, automatically learning which channels are more critical for specific tasks, and enhancing the model's sensitivity and expressive power for detailed features.

The counter module concludes with global pooling, a fully connected layer, and a decision output layer. Global pooling compresses the high‐dimensional features from the SENet module into a one‐dimensional feature vector. This step aggregates the statistical information of the entire audio segment, preparing for subsequent prediction of the number of sound sources. The fully connected layer maps the pooled feature vector to the classification space, with the output dimension equal to the number of possible source categories. Finally, the Argmax operation selects the category with the highest score as the final predicted number of sources *K*, and activates the corresponding decoder accordingly.

### Multi‐Head Decoder

Instead of a single general‐purpose decoder, we use a set of parallel decoders. For each input audio, the system first performs an argmax operation based on the Logits output by the counter module, dynamically selecting the number of predicted sound sources K and activating only the corresponding *i*th K decoder. The selected decoders generate soft masks using the Sigmoid activation function $M\in {\left[0,1\right]}^{k\times N_{\mathrm{enc}}\times L}$. These masks are continuous values and are applied to the encoder output through element‐wise multiplication w to isolate sound source features. i The reconstructed waveform of the *i*th sound source ${\hat{s}}_{i}$ is as follows:

$${\hat{s}}_{i}=\text{TransConv}1D\left(w⊙M_{i}\right)$$

Here, ⊙ element‐wise multiplication is represented, and TransConv1D is a transposed convolutional layer used to perform the inverse operation of the encoder, upsampling and converting back to the time domain to reconstruct a K separated audio waveform.

### Model Experiment Setup

The training and testing environment for the bird song separation network is shown in Table A1.

**TABLE A1:** Training and testing environment of the birdsong separation network.

Environment	Setup (training)	Setup (testing)
GPU	NVIDIA GeForce RTX A5000	NVIDIA GeForce RTX A5000
CPU	Intel Core i7‐11700F	Intel Core i7‐11700F
RAM	25GB	25GB
Operating system	Windows 10 64 bit	Windows 10 64 bit
Software environment	Python 3.9.19, Pytorch 1.13 CUDA 11.7	
